# Description of three new types of aurantiactinomyxon actinospores (Myxozoa: Myxosporea) from the oligochaete *Aulophorus* sp. in Tasik Telabak, Malaysia

**DOI:** 10.1007/s00436-026-08739-6

**Published:** 2026-07-29

**Authors:** Nadhirah Syafiqah Suhaimi, Csaba Székely, Gábor Cech, Boglárka Sellyei, Muhammad Hafiz Borkhanuddin

**Affiliations:** 1 HUN-REN Veterinary Medical Research Institute, 1143. Budapest, Hungária krt. 21, Budapest, Hungary; 2 Doctoral School of Animal Biotechnology and Animal Science, Hungarian University of Agriculture and Life Sciences, Gödöllő, Hungary; 3 Faculty of Science & Marine Environment, Universiti Malaysia Terengganu, Kuala Nerus, 21030 Malaysia

**Keywords:** Myxozoa, Parasite, Invertebrate host, Aurantiactinomyxon, Morphology, 18S rDNA

## Abstract

During a 2024 survey for actinosporeans at Tasik Telabak, Malaysia, three distinct aurantiactinomyxon types were identified from different specimens of the oligochaete *Aulophorus* sp. and characterized through both morphological and molecular analyses. Morphometric measurements revealed notable differences among the three types, particularly in the length of their caudal processes, despite all three types being found in the same host species. Analysis of the 18S ribosomal DNA (rDNA) of these aurantiactinomyxon types showed no close matches to existing myxozoan sequences in GenBank. Phylogenetic analyses placed all three types within a myxobolid clade dominated by *Henneguya* spp.; aurantiactinomyxon type 1 formed a subclade with *Myxobolus miyairii* from *Silurus asotus*, while aurantiactinomyxon types 2 and 3 clustered at the base of a larger subclade comprising mainly *Henneguya* species that infect *Ictalurus punctatus* and related catfish species. This study represents the second report of myxosporean infections in Malaysian oligochaete communities.

**Supplementary Information:**

The online version contains supplementary material available at https://doi.org/10.1007/s00436-026-08739-6.

## Highlights

- Description of three novel aurantiactinomyxon types from Tasik Telabak, Malaysia.
- First report of myxosporean infection in freshwater oligochaetes belonging to the genus *Aulophorus*.
- Expansion of the known oligochaete host range for the aurantiactinomyxon group.
- First record of the freshwater oligochaete species *Aulophorus* sp. in Tasik Telabak.

**Supplementary Information:**

The online version contains supplementary material available at https://doi.org/10.1007/s00436-026-08739-6.

## Introduction

The life cycle of myxozoans (Cnidaria: Myxosporea) is known to involve two hosts: a vertebrate host, which is typically a fish (Dyková et al. 2007), although infections have also been reported in mammals, amphibians, reptiles, and birds; and an invertebrate host, which is usually an oligochaete, polychaete or bryozoan (Hallett et al. 2015). Infected vertebrate hosts release myxospores or (fish) malacospores that infect annelid and bryozoan hosts through faeces or decaying bodies, whereas invertebrate hosts release actinospores or malacospores that attach to the skin and gills of vertebrate hosts, eventually infecting them and developing within their target organs (Kent and Hedrick 1986; El-Matbouli et al. 1995; Özer and Wootten 2000; Bjork and Bartholomew 2010; Braun 2017).

Research on actinospores remains limited, with roughly 200 types reported to date (Rocha et al. 2024), compared to more than 3,000 myxosporean species described worldwide (Whipps et al. 2025). Actinospores are categorized into collective groups based on their morphology. Among these groups, the aurantiactinomyxon collective group is the most diverse, presently comprising 73 types (Rocha 2023; Rocha et al. 2024; Suhaimi et al. 2024a, b, 2025), followed by the triactinomyxon collective group with 63 types (Borkhanuddin et al. 2014; Székely et al. 2014; Xi et al. 2015; Rangel et al. 2015, 2016; Neetu et al. 2018; Suhaimi et al. 2024a, b; Batueva et al., 2025), and the raabeia collective group comprising 37 types (Rocha et al. 2019a; Suhaimi et al. 2024a, b, 2025). The remaining actinospore types belong to the less common collective groups, namely the neoactinomyxum, antonactinomyxon, helioactinomyxon, hexactinomyxon, hungactinomyxon, synactinomyxon, sphaeractinomyxon, seisactinomyxon, saccimyxon, and tetractinomyxon collective groups.

The aurantiactinomyxon collective group was first described by Janiszewska (1957), who characterized these actinospores as having a style-less body with three equally-sized processes that curve downwards and envelop the entire base of the body cavity. Later, Lom and Dyková (2006) refined the definition, describing aurantiactinomyxons as having an ellipsoidal body containing a sporoplasm with numerous secondary cells, three stout, semicircular, leaf-like valvular processes, and protruding polar capsules at its apex. Recently, Rocha (2023) updated the definition, describing aurantiactinomyxon as having spherical, subspherical, cylindrical or triangular actinospores with three polar capsules protruding from the apex and three equally-sized latero-posterior valvular processes that arise from the style-less actinospore body. These processes curve downward and taper to a rounded or pointed end, and may be leaf-like, propeller-like, digitiform or triangular. Under this revised definition, the guyenotia collective group, previously distinguished by digitiform processes, was merged with the aurantiactinomyxon collective group, bringing the total number of recognized aurantiactinomyxon types to 73, including the former guyenotia types (Rocha 2023).

The majority of aurantiactinomyxon types have been reported from freshwater oligochaetes belonging to the family Naididae, particularly *Branchiura sowerbyi*, but also from *Tubifex tubifex*, *Limnodrilus hoffmeisteri*, *Dero digitata*, *Lophochaeta ignota*, *Nais* sp. and *Ilyodrilus templetoni* (Rocha 2023; Rocha et al. 2024). Other aurantiactinomyxon types have been reported from oligochaetes of the family Lumbriculidae, including *Stylodrilus heringianus* (Marques 1984; Oumouna et al. 2003; Holzer et al. 2004; Marcucci et al. 2009) and *Lumbriculus variegatus* (McGeorge et al. 1997; Xiao and Desser 1998; Özer and Wootten 2001; Freeman and Kristmundsson 2018). In this study, we conducted a survey of the myxosporean fauna infecting freshwater oligochaete communities in Tasik Telabak, a freshwater lake in Terengganu, Malaysia. This survey was part of an ongoing effort to document actinosporean diversity and improve our understanding of the myxozoan life cycle in Malaysian freshwater ecosystems.

## Materials and methods

### Sample collection

A survey of actinosporean infections in oligochaetes was conducted at Tasik Telabak, located in the upstream region of Besut, Terengganu (5^◦^37′58.22″ N, 102^◦^28′44.52″ E). Superficial sediment (0–5 cm depth) was collected from the shallow parts of the lake in September and October 2024 using a shovel. The collected sediments were placed in 10 L buckets with a minimal amount of lake water (1.5–3.0 L) and transported to the Marine Science Laboratory of the Faculty of Science and Marine Environment at Universiti Malaysia Terengganu. Upon arrival, the buckets were fitted with an aeration system, regularly supplied with 1.5 mL of dechlorinated tap water, and kept at room temperature (28–32 °C). The following day, the oligochaetes were manually separated by placing small portions of sediment onto a transparent tray placed over a light panel. Individual organisms were transferred into individual wells of 48-well microtiter plates containing dechlorinated water. Larger oligochaetes, such as *Branchiura sowerbyi* and *Branchiodrilus* sp., were transferred to 24-well plates within 24 h of collection. All isolated oligochaetes were examined daily for actinospore release using a Leica DM IL LED inverted microscope (Leica, Wetzlar, Germany). The sediment stock was kept for approximately 2 months, until the end of October 2024. Oligochaetes were maintained in cell-well plates for up to one week, without food, to avoid the presence of interfering particles; during this time, dead individuals were replaced with new ones. Oligochaetes in the sediment stock were fed with ground fish pellets every two weeks. All isolated oligochaetes were identified using the dichotomous key of Timm (1999), while individuals releasing actinospores were subsequently confirmed through molecular analysis, as described in DNA isolation, PCR and sequencing section.

### Morphological examination

Several drops of water from the cell-well plates wells that contained fresh actinospores; moreover the oligochaetes releasing spores were examined under an Olympus CX33 microscope (Olympus Corporation, Japan). Actinospores and oligochaetes were preserved in 90% ethanol for molecular studies or fixed in 10% neutral buffered formalin (NBF) for subsequent morphological examination. Each positive (actinospore-containing) well was processed individually without pooling. Samples were transported to the Fish Pathology and Parasitology Laboratory at the HUN-REN Veterinary Medical Research Institute in Budapest, Hungary, where 10% NBF-fixed samples (oligochaetes and actinospores) were examined under high magnification and photographed using an Olympus SZX16 stereo microscope and an Olympus BX53 light microscope equipped with an Olympus DP74 digital camera (Olympus Corporation, Japan), respectively. Actinospore morphometry was determined following the guidelines of Lom et al. (1997) and using ImageJ software (http://imagej.nih.gov/ij). All measurements are presented as the mean and standard deviation (SD), with the range given in parentheses, in micrometers (µm).

### DNA isolation, PCR, and sequencing

DNA was extracted from fifteen actinospore and three oligochaete samples that had been preserved in 90% ethanol. Prior to extraction, the actinospore samples were centrifuged at 13,000 rpm for 15 min; the ethanol was removed by pipetting, and they were washed twice with 10 mM Tris-HCl (pH 8.5) to remove residual ethanol. Genomic DNA was isolated from both types of samples using the Geneaid Tissue Genomic DNA Mini Kit (Geneaid Biotech Ltd., Taiwan) according to the manufacturer’s instructions for animal tissue samples.

Amplification of the parasite 18S rDNA gene and the internal transcribed spacer region (ITS) (internal transcribed spacer 1–5.8 S rRNA gene–internal transcribed spacer 2) of infected oligochaetes was performed by PCR (polymerase chain reaction) using the primer sets listed in Table 1. PCR amplification was performed in 25 µL reaction volumes containing 1–2 µL of template DNA, 1× DreamTaq buffer (10×; Thermo Scientific), 0.2 mM dNTP mix (10 mM; Thermo Scientific), 1–1.25 U DreamTaq polymerase (5 U; Thermo Scientific), 12.5 pmol of each primer, and molecular grade water.

**Table 1:** Primers used for PCR amplification and sequencing of each actinospore type and oligochaete

Species	Primer	Application	Reference
Aurantiactinomyxon type 1	MyxospecF	PCR	Fiala (2006)
	18R	PCR	Whipps et al. (2003)
	Myxgen4F	PCR and sequencing	Diamant et al. (2004)
	ERIB10	PCR and sequencing	Barta et al. (1997)
	Myxgen4R	Sequencing	Køie et al. (2008)
	SphR	Sequencing	Eszterbauer and Székely (2004)
Aurantiactinomyxon type 2	MyxospecF	PCR and sequencing	Fiala (2006)
	18R	PCR	Whipps et al. (2003)
	ERIB1	PCR and sequencing	Barta et al. (1997)
	Myxgen4R	PCR and sequencing	Køie et al. (2008)
	ACT1F	Sequencing	Hallett and Diamant (2001)
	18E	Sequencing	Hillis and Dixon (1991)
Aurantiactinomyxon type 3	MyxospecF	PCR and sequencing	Barta et al. (1997)
	18R	PCR	Diamant et al. (2004)
	ERIB1	PCR and sequencing	Hillis and Dixon (1991)
	Myxgen4R	PCR and sequencing	Fiala (2006)
	Myxgen4F	Sequencing	Diamant et al. (2004)
	ACT1fr	Sequencing	Hallett and Diamant (2001)
	SphR	Sequencing	Eszterbauer and Székely (2004)
	18E	Sequencing	Hillis and Dixon (1991)
*Aulophorus* sp.	ITS-5	PCR and sequencing	White et al. (1990)
	ITS-4	PCR and sequencing	White et al. (1990)
	5.8mussF	Sequencing	Källersjö et al. (2005)
	5.8mussR	Sequencing	Källersjö et al. (2005)

All reactions were conducted using a Doppio Gradient Thermal Cycler (VWR) following the thermal profiles shown in Table 2. PCR products were checked by agarose gel electrophoresis, purified using a DNA Fragment Purification kit (InViTek GmbH, Berlin, Germany), and sequenced bidirectionally using a BigDye Terminator v3.1 Cycle Sequencing Kit (Applied Biosystems, Foster City, CA, USA). The samples were sent at Delta Bio 2000 Ltd., Szeged, Hungary for electrophoretic analysis on an ABI PRISM 3100 Genetic Analyser (Applied Biosystems), using the primers listed in Table 1.

**Table 2:** PCR conditions for 18 S rDNA and ITS amplification using different primer pairs

Primer pair	Initial denaturation	Cycles (denaturation, annealing, elongation)	Final elongation	Reference
MyxospecF-18R	94 °C, 3 min	35 cycles: 95 °C 30 s, 55 °C 45 s, 72 °C 90 s	72 °C, 7 min; elongation adjusted to 90 s	Liu et al. (2016)
Myxgen4F-ERIB10	94 °C, 3 min	35 cycles: 94 °C 45 s, 55 °C 50 s, 72 °C 1 min	72 °C, 10 min	Colunga et al. (2024)
ERIB1-Myxgen4R	94 °C, 3 min	35 cycles: 95 °C 45 s, 55 °C 60 s, 72 °C 2 min	72 °C, 7 min	Present study
ITS 5-ITS 4	95 °C, 5 min	35 cycles: 95 °C 30 s, 50 °C 30 s, 72 °C 90 s	72 °C, 8 min	Erséus et al. (2017)

### Phylogenetic analyses

The ambiguous nucleotides of 18S rDNA and ITS sequences obtained in this study were checked and corrected (if necessary) based on the chromatogram and assembled using Geneious Prime v.11.1 (Kearse et al. 2012). Sequence alignments were performed using the ClustalW algorithm (Thompson et al. 1994) in MEGA X (Kumar et al. 2018). The dataset for actinospore analysis included four newly generated sequences from this study, along with 46 reference sequences obtained from BLASTn searches, using a threshold of ≥ 85% sequence identity and query coverage. Sequences with lower identity (< 85%) or poor support were excluded. An outgroup, *Chloromyxum cristatum*, was selected based on its basal position in myxozoan phylogeny. For the oligochaete dataset, two new sequences from this study were analyzed together with 23 representative ITS sequences showing ≥ 79% identity, while sequences below this threshold were excluded. The outgroup, *Fridericia tuberosa*, was chosen based on its basal position in oligochaete phylogeny.

Phylogenetic trees were generated using Maximum Likelihood (ML) in MEGA X with 1,000 bootstrap replicates. Alignment gaps were treated using partial deletion with a 75% site coverage cut-off. The analyses were performed using the GTR + G + I model based on the Akaike information criterion (AIC). Clades with ML bootstrap support values ≥ 70% were considered well-supported. The resulting phylogenetic trees were visualized in MEGA X and FigTree v. 1.4.4 (Rambaut 2018), and graphically edited with Inkscape (Free Software Foundation, Inc., MA, USA). Genetic distances were calculated from the same datasets using the *p*-distance method in MEGA X.

## Results

### Oligochaete collection and identification

**Fig. 1:**
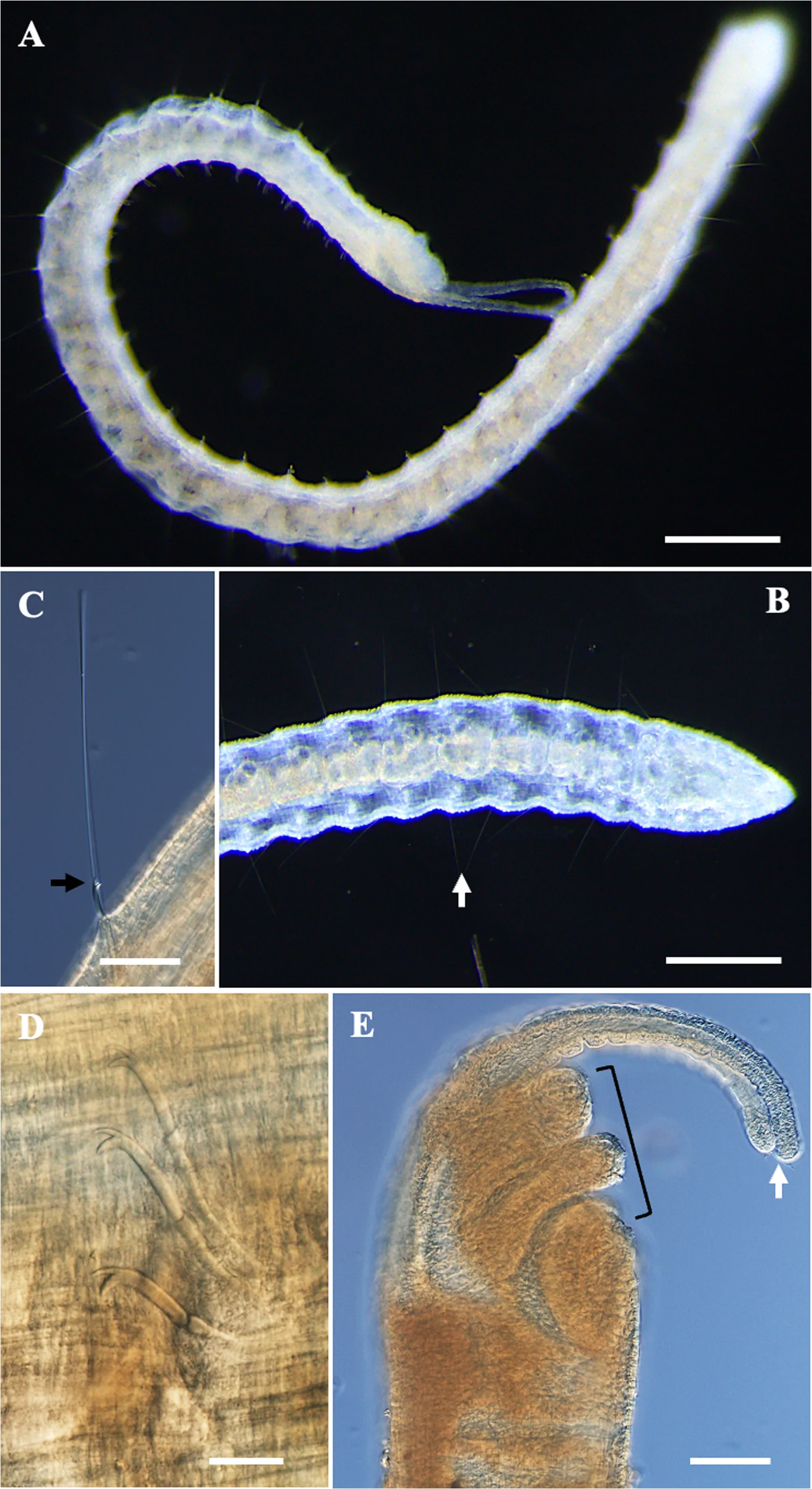
(**A**) *Aulophorus* sp. (**B**) Dorsal view showing one hair chaeta (black arrow) and (**C**) one bifid needle chaeta (white arrow). (**D**) Detail view displaying three ventral chaetae. (**E**) Posterior end exhibiting a pair of finger-like, non-contractile palps (white arrow) and three pairs of branchial discs (black bracket). Scale bars represent 50 μm, except (A–B) 200 μm and (**D**) 20 μm

**Fig. 2:**
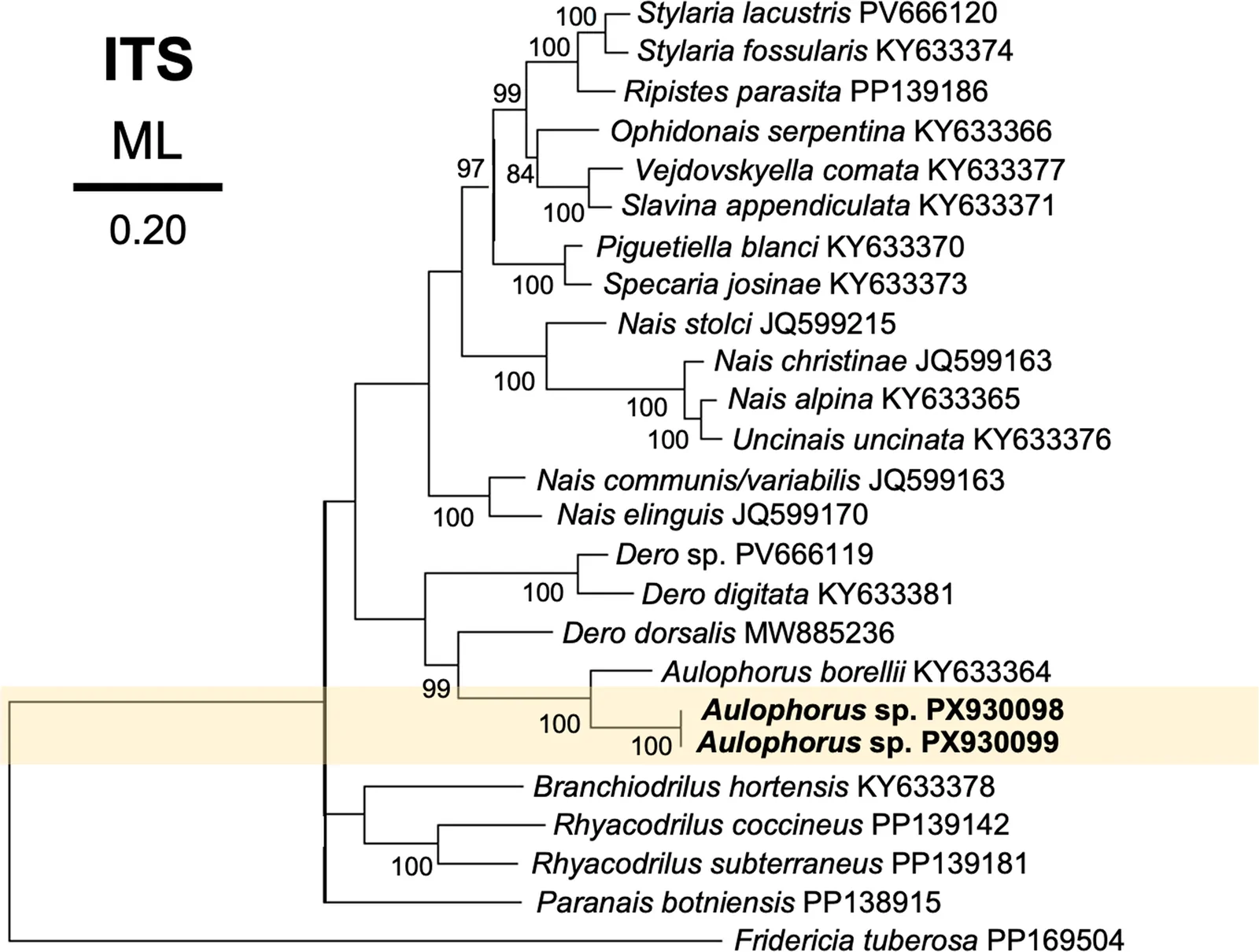
Maximum likelihood (ML) phylogenetic tree of ITS sequences of *Aulophorus* sp. and related species. *Fridericia tuberosa* was used as the outgroup. Nodal supports are indicated for ML at 1000 replicates. Only values with ≥ 70% bootstrap (BS) support are presented. The names of the oligochaetes appear before GenBank accession numbers. Sequences obtained in the present study are shown in bold within colored boxes. The scale bar indicates the expected number of substitutions per site

In September to October 2024, 1,354 oligochaetes were isolated from sediment collected at Tasik Telabak, Terengganu. Eight distinct oligochaete types were observed: *Branchiodrilus* (*n* = 235), *Branchiura* (*n* = 104), *Tubifex* (*n* = 148), *Bothrioneurum* (*n* = 73), *Aulodrilus* (*n* = 86), *Dero* (*n* = 98), *Limnodrilus* (*n* = 120), and *Aulophorus* (*n* = 490). Myxosporean infections were detected exclusively in individuals belonging to the genus *Aulophorus* (Naididae). The infected host was initially identified to the genus level based on morphological characteristics and subsequently confirmed using nuclear ITS sequence data. The infected oligochaetes were identified as members of the genus *Aulophorus*, based on the morphological characteristics described by Timm (1999), namely one hair chaeta and one needle chaeta on the dorsal side, three to four ventral chaetae per bundle with a marginally longer upper tooth (Fig. 1A–D), and non-contractile palps with three pairs of posterior gills (Fig. 1E). A BLAST search of 913 bp ITS sequences (PX930098 and PX930099) obtained from the infected oligochaetes showed 83.1% similarity to *Dero dorsalis* (MW885236) and 79.7% similarity to *Aulophorus borelli* (KY633381) (Table 3). Morphologically, *Aulophorus* differs from *Dero* by possessing non-contractile palps, whereas *Dero* possesses contractile posterior gills (Timm 1999). Due to the relatively low sequence similarity and limited sequence length, identification of the infected oligochaetes was limited to the genus level. However, phylogenetic analyses further indicated that the infected oligochaetes were more closely related to *A. borelli*, with maximum bootstrap support, than to *D. dorsalis* (Fig. 2). Given the distinct phylogenetic grouping and morphological features, all the host specimens were considered *Aulophorus* sp.

**Table 3:** Genetic *p*-distance and sequence similarities (%) for the ITS region of studied oligochaetes and closely related oligochaete species. The sequences from the present study are in bold

Sequence	Genetic distance	Sequence similarities (%)
*Aulophorus* sp. PX930099		
*Aulophorus* sp. PX930098	0.000	100.0
*Dero dorsalis* MW885236	0.134	86.6
*Aulophorus borellii* KY633364	0.155	85.5
*Nais christinae* JQ599163	0.222	77.8
*Dero digitata* KY633381	0.280	72.0
*Dero* sp. PV666119	0.294	70.6

### Overall prevalence

Actinosporean infection was detected by observing the release of actinospores from 15 *Aulophorus* individuals, resulting in an overall prevalence of infection of 1.1% (15/1354) among all oligochaetes examined and a prevalence of 3.0% (15/490) among all *Aulophorus* specimens. Three distinct actinospore types, all belonging to the aurantiactinomyxon collective group, were released by infected hosts within 24 h of individual isolation and continued to be released for the following six days. Morphological and molecular analyses identified them as novel types.

### Description of novel actinospore types

#### Aurantiactinomyxon type 1 (Figs. 3A and 4A)

**Fig. 3:**
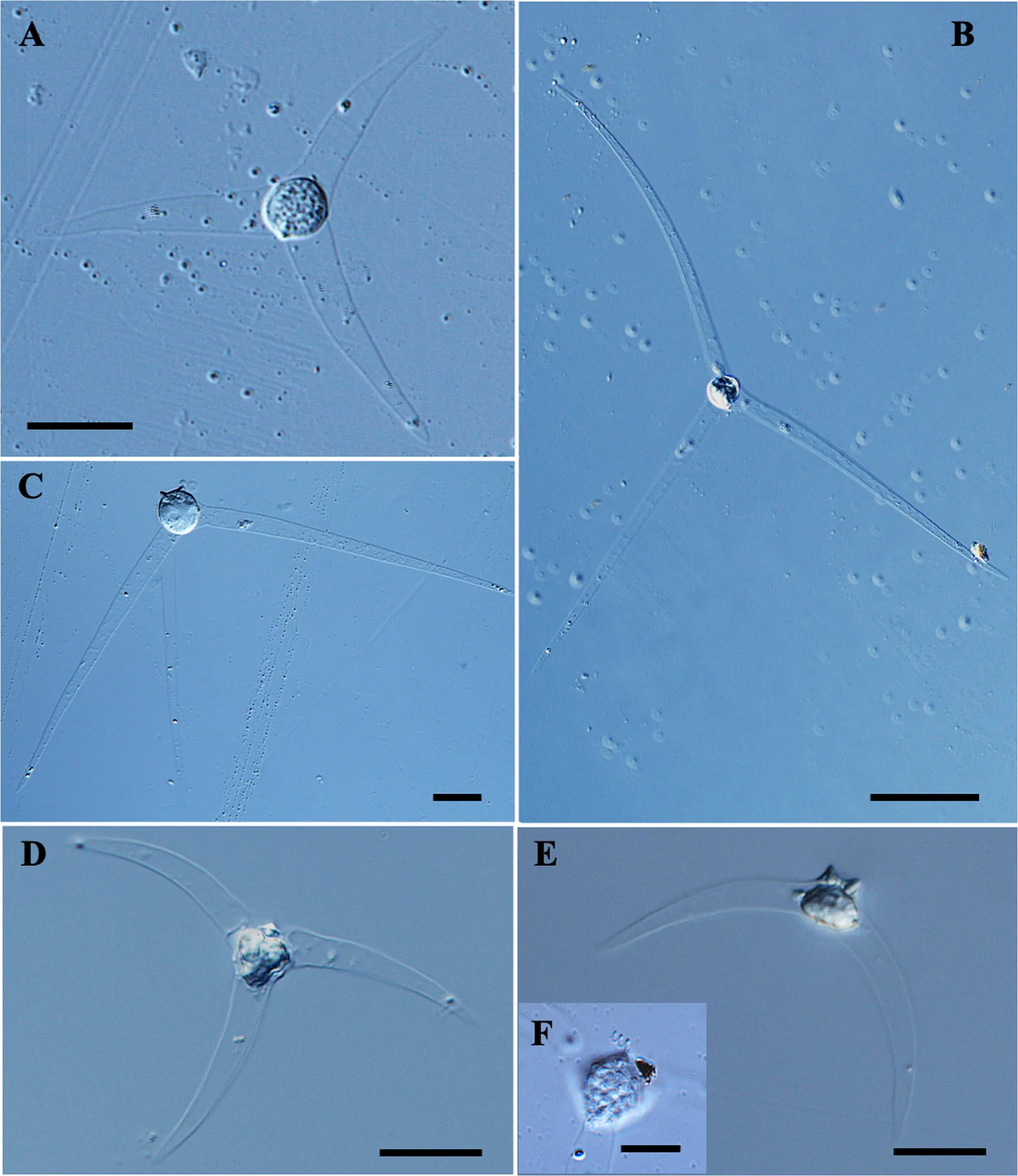
Aurantiactinomyxon types released from *Aulophorus* sp. (**A**) Aurantiactinomyxon type 1 in apical view. (**B**) Aurantiactinomyxon type 2 in apical view. (**C**) Aurantiactinomyxon type 2 in sutural view. (**D**) Aurantiactinomyxon type 3 in apical view. (**E**) Aurantiactinomyxon type 3 in sutural view. (**F**) Actinospore body showing an extruded polar tubule with three coils. Scale bars represent 20 μm, except (**C**) 50 μm and (F) 10 μm

**Fig. 4:**
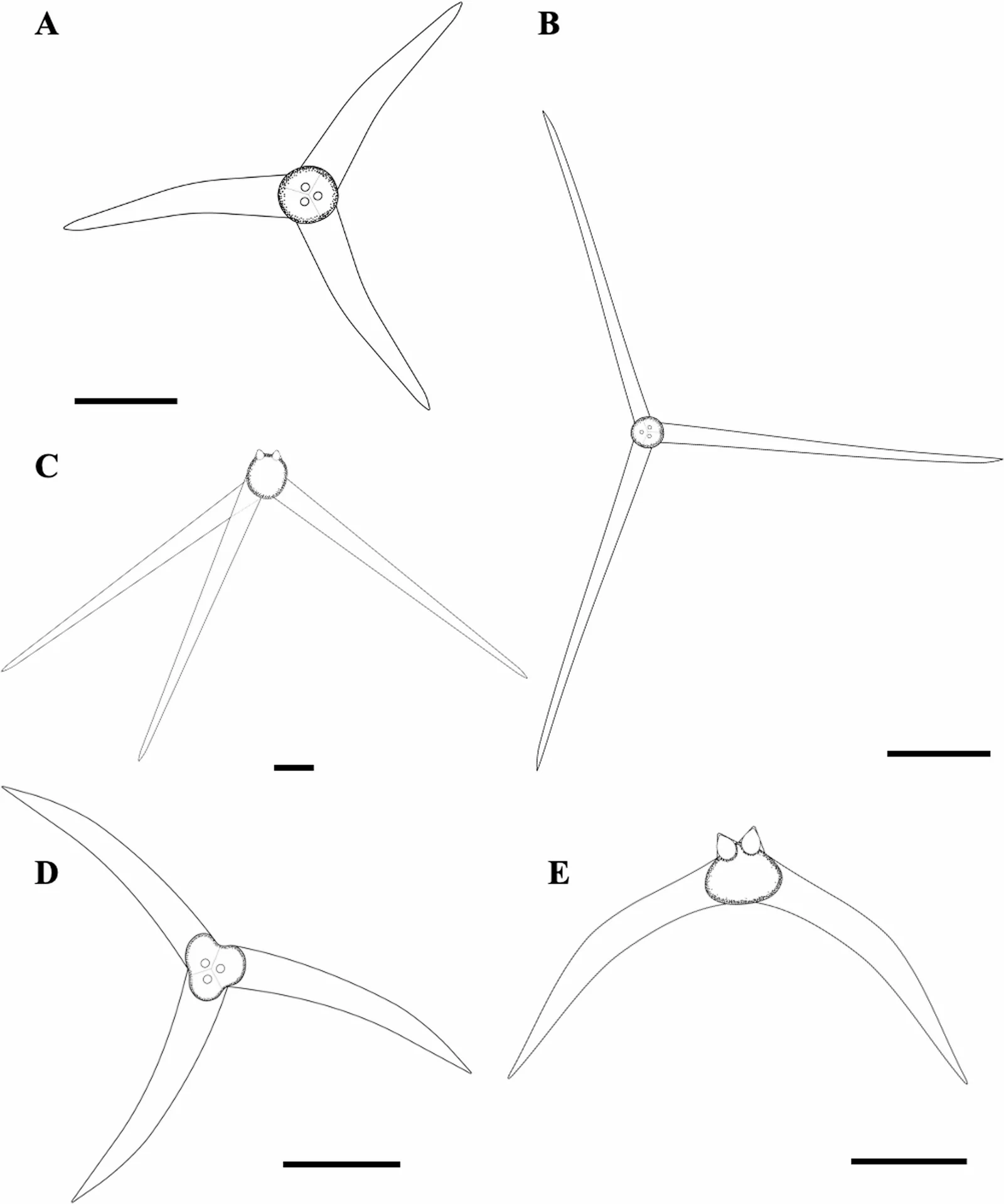
Schematic drawings of Aurantiactinomyxon types from *Aulophorus* sp. (**A**) Aurantiactinomyxon type 1 in apical view. (**B**) Aurantiactinomyxon type 2 in apical view. (**C**) Aurantiactinomyxon type 2 in sutural view. (**D**) Aurantiactinomyxon type 3 in apical view. (**E**) Aurantiactinomyxon type 3 in sutural view. Scale bars represent 20 μm, except (**C**) 50 μm

Spore body: Spherical, 10.0 ± 0.9 (8.5–11.1) µm in diameter. Caudal processes: Equally sized, elongated, tapering to pointed ends, 39.7 ± 2.2 (34.4–43.4) µm long, 6.8 ± 0.4 (6.0–7.5) µm wide at the base. Polar capsules: Three, equally sized, pyriform, protruding from the apex of the actinospore body, 2.6 (2.6–2.7) µm long, 1.6 ± 0.1 (1.5–1.8) µm wide (*n* = 3). Polar tubule: Coiled three times. Sporoplasm: 32 secondary cells. Measurements from 26 formalin-fixed actinospores.

Host: Species of the genus *Aulophorus* Schmarda, 1861 (Family: Naididae).

Infection site: Intestinal epithelium.

Prevalence: 0.2% (1 infected in 490 *Aulophorus* individuals examined).

Type material: Series of phototypes deposited in the collection of Fish Pathology and Parasitology Research Team, HUN-REN Veterinary Medical Research Institute, Budapest, Hungary.

Molecular data: Partial 18S rDNA sequence with 1,447 base pairs, deposited in GenBank under accession number PX930095. Pairwise distance estimation indicated the highest similarity of 86.1% to *Henneguya* sp. (PP852214) and 85.7% to *Henneguya quelen* (MH230064) (Table 4).

**Table 4:** Genetic *p*-distance and sequence similarities (%) for the 18S rDNA of aurantiactinomyxon types and closely related myxozoan species. Taxa were selected based on BLAST results showing ≥ 85% identity and high query coverage with 75% cut-off. The sequences from the present study are in bold

Sequence	Genetic distance	Sequence similarities (%)
Aurantiactinomyxon type 1 PX930095		
*Henneguya* sp. PP852214	0.139	86.1
*Henneguya quelen* MH230064	0.143	85.7
*Henneguya sutherlandi* MZ905346	0.151	84.9
*Henneguya multiradiatus* MT415832	0.151	84.9
Raabeia type KF263539	0.154	84.6
*Henneguya chesapeakensis* PV114887	0.154	84.6
*Henneguya pellis* FJ468488	0.155	84.5
*Myxobolus miyairii* KT001495	0.156	84.4
Aurantiactinomyxon type 2 PX930094		
Aurantiactinomyxon type 3 PX930097	0.058	94.2
Aurantiactinomyxon type 3 PX930096	0.059	94.1
*Henneguya* sp. PP852214	0.096	90.4
Aurantiactinomyxon type 3 PX930097		
Aurantiactinomyxon type 3 PX930096	0.001	99.9
Aurantiactinomyxon type 2 PX930094	0.059	94.1
*Henneguya* sp. PP852214	0.093	90.7

Remarks: The morphometry of aurantiactinomyxon type 1 does not specifically match any previously described aurantiactinomyxon type. It is morphologically most similar to aurantiactinomyxon type 1 of *Thelohanellus hovorkai* (Suhaimi et al. 2026), but it is smaller overall, with the only shared feature being the presence of 32 secondary cells. Morphometrically, it most closely resembles the aurantiactinomyxon type 2 as reported by Milanin et al. (2018), particularly in polar capsule width. However, the actinospore body diameter and caudal process width of aurantiactinomyxon type 1 are smaller, while the caudal process and polar capsule length are longer than those reported for aurantiactinomyxon type 2 of Milanin et al. 2018 (Table 5).

**Table 5:** Comparison of morphometric measurements of aurantiactinomyxon types from the present study and morphologically similar types reported in the literature

Aurantiactinomyxon type	Host	SBD	CPL	CPW	PCL	PCW	SC	Fixation type	Reference
Aurantiactinomyxon type 1	***Aulophorus*** **sp.**	**10.0 ± 0.9** **(8.5–11.1)**	**39.7 ± 2.2** **(34.4–43.4)**	**6.8 ± 0.4** **(6.0–7.5)**	**2.6** **(2.6–2.7)**	**1.6 ± 0.1** **(1.5–1.8)**	**32**	**Formalin**	**Present study**
Aurantiactinomyxon type 2	*Pristina synclites*	11.2 (10.2–11.9)	30.4 (25.0–33.3)	7.0 (5.4–7.7)	1.5 (1.3–1.8)	1.5 (1.3–1.8)	**–**	Fresh	Milanin et al. (2018)
Aurantiactinomyxon type 2	***Aulophorus*** **sp.**	**17.2 ± 1.2** **(15.8–20.0)**	**153.6 ± 7.2** **(144.2–171.0)**	**8.4 ± 0.7** **(7.1–9.9)**	**5.1 ± 0.3** **(4.1–6.4)**	**2.6 ± 0.4** **(2.0–3.3)**	**–**	**Formalin**	**Present study**
Aurantiactinomyxon type	*B. sowerbyi*	19.7 (18.9–21.9)	170.8 (167.5–176.3)	12.9 (11.2–13.5)	3.1 (2.9–3.2)	1.7 (1.5–1.9)	64	Fresh	Xi et al. (2013)
Aurantiactinomyxon type 3	*Tubifex tubifex*	L: 24 (23.4–24.9) W: 21.8 (20.3–23.4)	114.5 (101.4–124.8)	–	4.0	3.2	32	Fresh	Özer et al. (2002)
Aurantiactinomyxon type 3	***Aulophorus*** **sp.**	**11.7 ± 0.7** **(10.5–13.2)**	**44.3 ± 2.9** **(40.4–49.3)**	**6.3 ± 0.5** **(5.5–7.6)**	**3.2 ± 0.2** **(2.7–3.5)**	**2.0 ± 0.2** **(1.5–2.4)**	**–**	**Formalin**	**Present study**
Aurantiactinomyxon type	*Bothrioneurum* sp.	8.8 ± 0.7 (7.6–10.1)	36.7 ± 2.7 (30.7–43.8)	4.9 ± 0.6 (3.9–6.2)	3.6 ± 0.3 (3.0–4.3)	1.3 ± 0.2 (1.1–1.9)	**–**	Ethanol	Suhaimi et al. (2024a)
Aurantiactinomyxon type of *Henneguya exilis*	*Dero digitata*	11.7 ± 0.8 (10.2–13.3)	42.4 ± 2.4 (37.6–46.2)	6.5 ± 0.8 (5.2–8.5)	–	–	–	Fresh	Rosser et al. (2014)

All measurements are in µm and ’–’ indicates no data. SBD: actinospore body diameter, *CPL* caudal processes length, *CPW* caudal processes width, *PCL* polar capsule length, *PCW* polar capsule width, *SC* number of secondary cells, *D* diameter, *L* length, *W* width

#### Aurantiactinomyxon type 2 (Figs. 3B–C and 4B–C)

Spore body: Subspherical in both apical and side views, 17.2 ± 1.2 (15.8–20.0) µm in diameter. Caudal processes: Three, equally sized, elongated, tapering to pointed ends, 153.6 ± 7.2 (144.2–171.0) µm long, 8.4 ± 0.7 (7.1–9.9) µm wide at the base. Polar capsules: Pyriform, protruding from the apex of the actinospore body, 5.1 ± 0.5 (4.1–6.4) µm long, 2.6 ± 0.4 (2.0–3.3) µm wide (*n* = 25). Polar tubule: Undetermined number of coils. Sporoplasm: Undetermined number of secondary cells. Measurements from 33 formalin-fixed actinospores derived from a single individual.

Host: Species of the genus *Aulophorus* Schmarda, 1861 (Family: Naididae).

Infection site: Intestinal epithelium.

Prevalence: 0.4% (2 infected in 490 *Aulophorus* individuals examined).

Type material: Series of phototypes deposited in the collection of Fish Pathology and Parasitology Research Team, HUN-REN Veterinary Medical Research Institute, Budapest, Hungary.

Molecular data: Partial 18S rDNA sequence with 1,780 base pairs, deposited in GenBank under the accession number PX930094. Pairwise distance estimation indicated the highest similarity of 94.2% to aurantiactinomyxon type 3 (PX930096, PX930097) described in this study (Table 4).

Remarks: The morphology of aurantiactinomyxon type 2 does not match any previously described aurantiactinomyxon types. According to previous studies, only the aurantiactinomyxon type reported by Xi et al. (2013) and aurantiactinomyxon type 3 of Özer et al. (2002) exhibit caudal process lengths that exceed 100 μm. However, the present aurantiactinomyxon type 2 differs from both in all measured characteristics and infects a different host species. Based on caudal process length and width, and polar capsule width, aurantiactinomyxon type 2 ranks as the second largest among the types mentioned above. In contrast, it possesses the longest polar capsule length and the smallest spore body compared to the aurantiactinomyxon type of Xi et al. (2013) and aurantiactinomyxon type 3 of Özer et al. (2002) (Table 5). Further infections from other infected individuals were examined, and morphometric data from 12 actinospores are provided below. The spore body was 16.7 ± 0.7 (15.9–17.8) µm in diameter. Caudal processes were three, equally sized, elongated, tapering to pointed ends, measuring 155.2 ± 7.6 (144.2–171.0) µm in length and 8.3 ± 0.8 (7.1–9.5) µm in width at the base. Polar capsules were pyriform and protruded from the apex of the actinospore body, measuring 5.3 ± 1.0 (4.5–6.4) µm in length and 2.6 ± 0.5 (2.1–3.1) µm in width (*n* = 3). The number of polar tubule coils and the number of secondary cells in the sporoplasm were undetermined. These measurements provide additional information on intra- and inter-type size variation.

#### Aurantiactinomyxon type 3 (Figs. 3D–F and 4D–E)

Spore body: Pyramidal in both apical and side views, 11.7 ± 0.7 (10.5–13.2) µm in diameter. Caudal processes: Three, equally sized, elongated, tapering to pointed ends, 44.3 ± 2.9 (40.4–49.3) µm long, 6.3 ± 0.5 (5.5–7.6) µm wide at the base. Polar capsules: Pyriform, protruding from the apex of the actinospore body, 3.2 ± 0.2 (2.7–3.5) µm long, 2.0 ± 0.2 (1.5–2.4) µm wide (*n* = 25). Polar tubule: Coiled three times (Fig. 3F). Sporoplasm: Undetermined number of secondary cells. Measurements from 30 formalin-fixed actinospores derived from a single individual.

Host: Species of the genus *Aulophorus* Schmarda, 1861 (Family: Naididae).

Infection site: Intestinal epithelium.

Prevalence: 2.4% (12 infected in 490 *Aulophorus* individuals examined).

Type material: Series of phototypes deposited in the collection of Fish Pathology and Parasitology Research Team, HUN-REN Veterinary Medical Research Institute, Budapest, Hungary.

Molecular data: Two nearly identical 18S rDNA sequences of aurantiactinomyxon type 3 (99.9%; 1838 and 1939 bp) were deposited in GenBank under the accession numbers PX930097 and PX930096. Pairwise distance estimation indicated the highest similarity of 94.1% to the aurantiactinomyxon type 2 (PX930094) described in this study and 90.7% to *Henneguya* sp. (PP852214) (Table 4).

Remarks: The morphology of aurantiactinomyxon type 3 does not match any previously described aurantiactinomyxon types (Table 5). Its morphology more closely resembles that of the aurantiactinomyxon type described by Suhaimi et al. (2024a), which was also collected from Tasik Telabak. However, significant differences were observed in morphometry, with the present aurantiactinomyxon type 3 being larger in most dimensions. Notably, the aurantiactinomyxon type reported by Suhaimi et al. (2024a) exhibited prominent, elongated pyriform polar capsules protruding from the anterior of the actinospore body, distinguishing it from aurantiactinomyxon type 3. Morphometrically, aurantiactinomyxon type 3 most closely resembles the aurantiactinomyxon type of *Henneguya exilis* (Rosser et al. 2014); however, it differs in spore body dimensions, and no measurements were provided for the polar capsule length, width, or secondary cells. Further infections from other hosts were examined, and morphometric data from two actinospores per host (*n* = 11) are provided in Supplementary Table 1.

### Phylogeny

The assembled nucleotide sequences obtained from the amplification of the actinospore 18S rDNA region were 1447, 1780, and 1838–1939 bp long. The phylogenetic alignment of all the sequences contained 2,044 nucleotide positions including 1,075 conserved and 902 variable nucleotide sites.

**Fig. 5:**
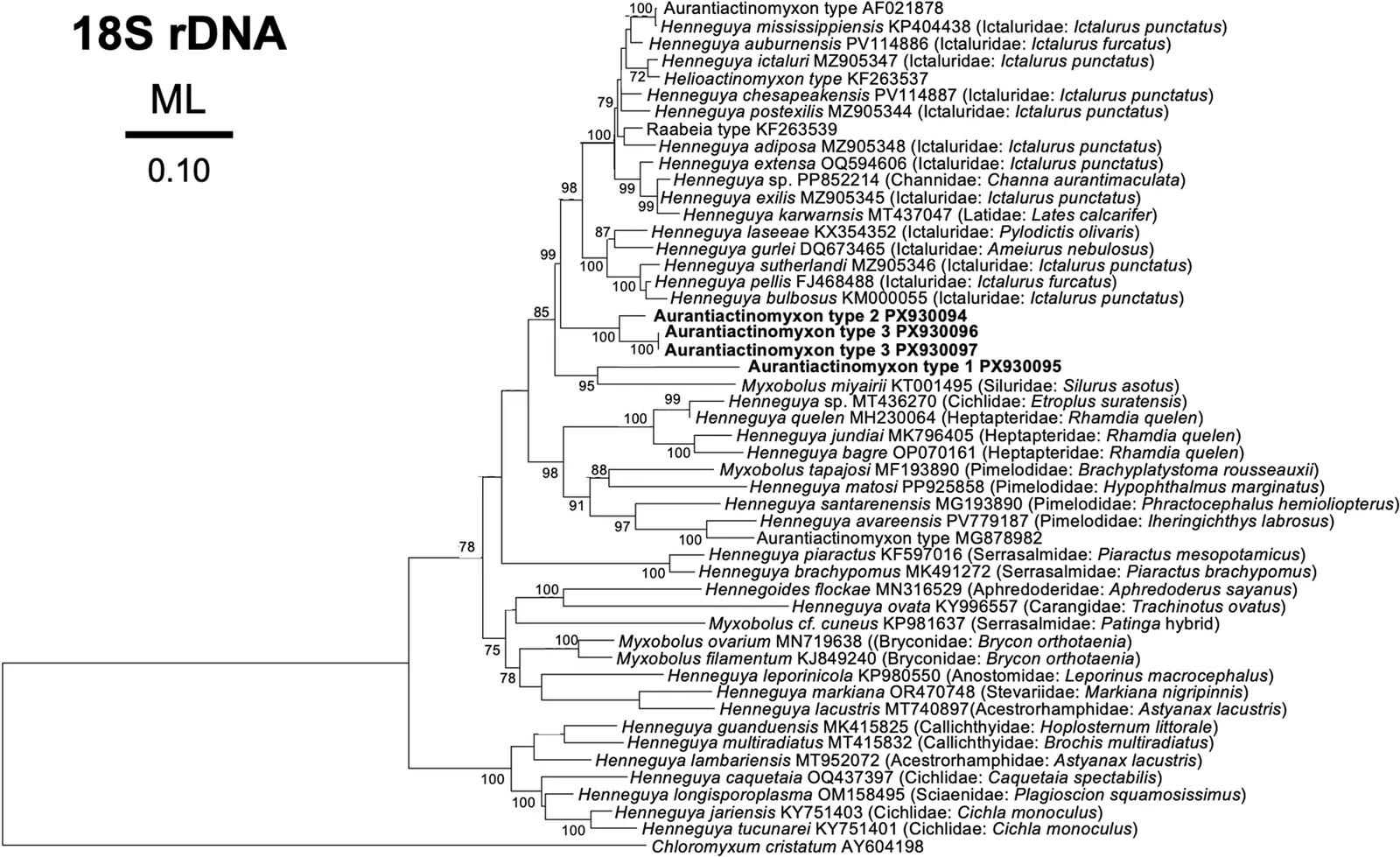
Maximum likelihood (ML) phylogenetic tree of 18S rDNA sequences of aurantiactinomyxon type 1, aurantiactinomyxon type 2, aurantiactinomyxon type 3 and related species. *Chloromyxum cristatum* was used as an outgroup. Nodal supports are indicated for ML at 1000 replicates. Only values with ≥ 70% bootstrap (BS) support are presented. The names of the parasite species appear before GenBank accession numbers. Host family and species included within parentheses. The scale bar indicates the expected number of substitutions per site

Phylogenetic analysis of the 18S rDNA dataset revealed that aurantiactinomyxon type 1 was closely related to *Myxobolus miyairii* (KT001495), being positioned at the base of a larger clade comprising mainly *Henneguya* sequences, together with several raabeia and aurantiactinomyxon types, including those described in the present study, with high bootstrap support. The aurantiactinomyxon types 2 (PX930094) and 3 (PX930096, PX930097) identified in this study were most closely related to each other, with maximum bootstrap support (Fig. 5).

## Discussion

During this study, three actinospore types belonging to the aurantiactinomyxon collective group were identified and described from *Aulophorus* sp. in Tasik Telabak, Malaysia. This finding resulted from ongoing surveys of myxosporeans in annelid communities in Tasik Telabak, and represents only the second report documenting actinospore stages in Malaysia (Suhaimi et al. 2024a). Myxospore stages corresponding to these actinospore types are yet to be determined.

Most known aurantiactinomyxon types infect naidid oligochaetes in freshwater or marine environments (Rocha 2023; Rocha et al. 2024; Suhaimi et al. 2024b, 2025). In this study, we identified a species of the genus *Aulophorus* (Naididae) as a new freshwater host for the aurantiactinomyxon, representing the first reported myxosporean infection in this genus worldwide. Recently, Suhaimi et al. (2024a) identified species of the genus *Bothrioneurum* as potential hosts for the aurantiactinomyxon group. Additionally, these authors found raabeia and triactinomyxon types from *Aulodrilus acutus* and *Branchiodrilus* sp. in Tasik Telabak, Malaysia. This suggests that this water body harbors diverse oligochaete fauna, many of which are poorly studied and may serve as hosts for myxozoans. The genus *Aulophorus* is widely distributed throughout Southeast Asia, including Cambodia (Ohtaka et al. 2011), Indonesia (Ohtaka et al. 2015), Malaysia (Hettige et al. 2021), and Vietnam (Gusakov et al. 2023), as well as India (Vineetha et al. 2015), Brazil (Rodrigues et al. 2015), and West Africa (Martin et al. 2017). Based on our findings from Malaysia, these common oligochaetes appear to be susceptible to myxosporean infection, thereby broadening the known range of annelid hosts.

The overall prevalence of myxosporean infection in the oligochaetes examined was 1.1%, which is consistent with previous reports for other aurantiactinomyxon types occurring in wild environments (Xiao and Desser 1998; El-Mansy et al. 1998a, b; Székely et al. 2000, 2003, 2004; Xi et al. 2015; Zhao et al. 2016; Milanin et al. 2017; Rocha et al. 2019b, 2024). Additionally, the prevalence of infected oligochaetes from the genus *Aulophorus* was 3.0%. This aligns with a previous finding by El-Mansy et al. (1998b), who reported a 3.0% prevalence of aurantiactinomyxon types infecting *Limnodrilus hoffmeisteri* in Lake Balaton, Hungary. A recent study by Suhaimi et al. (2024a) reported a similar prevalence of infection (2.7%) by aurantiactinomyxon from *Bothrioneurum* sp. in Tasik Telabak, though no infections were detected in *Aulophorus* spp. examined between July and August 2023, corresponding to the dry season.

In contrast, infections were observed in *Aulophorus* sp. in the present study, in which oligochaetes were collected from September to October 2024, coinciding with the inter-monsoon period in Malaysia. This difference may be explained by variations in annelid infection prevalence driven by differences in the vertebrate host community. For example, shifts in fish abundance, density, or migration patterns during inter-monsoon periods could increase myxospore dispersal and encounter rates with specific oligochaetes such as *Aulophorus* sp., which may be more abundant or susceptible under these conditions (Koel et al. 2010; Alexander et al. 2015). In addition, only mature infections resulting in spore release were detected and measured. As actinospore development may take several weeks to months, the observed infections may reflect earlier exposure rather than contemporaneous transmission patterns. Therefore, infections may have been established prior to the inter-monsoon period but only became detectable during this time, suggesting that transmission could have occurred before or during the transition into the inter-monsoon period. This may explain why Suhaimi et al. (2024a) did not detect infections during the dry season.

According to current literature, aurantiactinomyxon collective group is one of the most diverse collective groups, comprising 73 known types (Rocha et al., 2024; Rocha et al. 2024; Suhaimi et al. 2024a, b, 2025). In this study, three novel aurantiactinomyxon types were identified from a single species, highlighting previously undocumented myxosporean diversity associated with *Aulophorus* sp. as an invertebrate host. None of the aurantiactinomyxon types described here exhibited morphological or morphometric resemblance to any previously reported aurantiactinomyxon types. However, it should be noted that all morphometric measurements in this study were obtained from spores fixed in 10% neutral buffered formalin, which may cause slight shrinkage or distortion of structures and potentially influence fine-scale comparisons with descriptions based on fresh material. Compared to each other, all three types showed significant differences across all morphometric measurements, including the length of their caudal processes. Aurantiactinomyxon type 2 exhibited the longest caudal processes among the three and ranked as the second largest among all previously reported aurantiactinomyxon types, following the aurantiactinomyxon type of Xi et al. (2013) from *B. sowerbyi*. In contrast, aurantiactinomyxon type 1 possessed the shortest caudal processes among the three, and its morphology closely resembled that of the aurantiactinomyxon type of *T. hovorkai* described by Suhaimi et al. (2026), sharing a similar number of secondary cells but being smaller overall. In turn, aurantiactinomyxon type 3 more closely resembled the aurantiactinomyxon type of Suhaimi et al. (2024a), that was reported from a different host (*Bothrioneurum* sp.) in Tasik Telabak.

Previously, myxozoan identification relied solely on spore morphology. However, molecular research has demonstrated that it can be misleading, especially when actinospores exhibit overlapping or similar features and infect the same or closely related hosts. For example, studies by Hallett et al. (2002); Eszterbauer et al. (2006); Zhao et al. (2016); Rocha et al. (2019b) have reported genetically identical actinospores from the aurantiactinomyxon, neoactinomyxum, and sphaeractinomyxon collective groups displaying notable variations in spore form.

In the present study, genetic analyses confirmed that all analyzed aurantiactinomyxon types represented distinct species. Phylogenetic analyses revealed that all three aurantiactinomyxon types were positioned within a myxobolid clade dominated by *Henneguya* spp. Aurantiactinomyxon type 2 and aurantiactinomyxon type 3 clustered with several species of *Henneguya* supported by a bootstrap value of 99%, particularly those infecting *Ictalurus punctatus* from the USA and China, as well as related catfish species such as *Pylodictis olivaris* and *Ameiurus nebulosus*. This relationship suggests that both aurantiactinomyxon types may represent the actinospore stages of *Henneguya* species that infect Malaysian catfish or related catfish hosts, with the only exceptions being *Channa aurantimaculata* and *Lates calcarifer*. In contrast, aurantiactinomyxon type 1 formed a subclade with *Myxobolus miyairii* from *Silurus asotus*, another siluriform fish that is phylogenetically distinct from ictalurid catfishes. However, there are currently no known aurantiactinomyxon types that correspond to the genus *Myxobolus*. The genera *Myxobolus* and *Henneguya* commonly appear intermixed in phylogenetic trees and do not form monophyletic clades (Fiala 2006; Liu et al. 2010; Gupta et al. 2021), making the position of aurantiactinomyxon type 1 alongside *M. miyairii* consistent with established patterns in actinosporean-myxosporean relationships.

In conclusion, three aurantiactinomyxon actinospore types were described via morphological and molecular analyses. All were collected from the identical oligochaete host species belonging to the genus *Aulophorus* (family Naididae) in Tasik Telabak, Malaysia. This study provides the first molecular characterization of aurantiactinomyxon types from Malaysian annelids, recognizing *Aulophorus* sp. as a previously unreported intermediate host. To fully elucidate the incomplete life cycles of the three aurantiactinomyxon types described in this study, further comprehensive surveys of fish populations in Tasik Telabak are needed, with particular attention to Malaysian catfish or related catfish hosts from the genera *Ictalurus* or *Silurus*, which may serve as potential hosts.

## Supplementary information

Below is the link to the electronic supplementary material.

Supplementary figure 6 (PNG 450 KB)

Supplementary Material 2

## Data Availability

DNA sequence data generated in this study have been deposited in GenBank under accession numbers PX930094–PX930099.

## References

[CR1] Alexander JD, Kerans BL, El-Matbouli M, Hallett SL, Stevens L (2015) Annelid-myxosporean interactions. In: Okamura B, Gruhl A, Bartholomew JL (eds) Myxozoan evolution, ecology and development. Springer International Publishing, Cham, pp 217–234

[CR2] Barta JR, Martin DS, Liberator PA, Dashkevicz M, Anderson JW, Feighner SD, Elbrecht A, Perkins-Barrow A, Jenkins MC, Danforth HD, Ruff MD, Profous-Juchelka H (1997) Phylogenetic relationships among eight *Eimeria* species infecting domestic fowl inferred using complete small subunit ribosomal DNA sequences. J Parasitol 83:262–271. 10.2307/3284453 9105308

[CR3] Batueva MDD, Abasheev RY (2025) The first report on the occurrence of an actinosporean stage (Myxozoa) in oligochaetes in the coastal zone of Lake Baikal. J Parasit Dis 1–7. 10.1007/s12639-025-01781-8 PMC12399461 40901416

[CR4] Bjork SJ, Bartholomew JL (2010) Invasion of *Ceratomyxa shasta* (Myxozoa) and comparison of migration to the intestine between susceptible and resistant fish hosts. Int J Parasitol 40:1087–1095. 10.1016/j.ijpara.2010.03.005 20385137

[CR5] Borkhanuddin MH, Cech G, Molnár K, Németh S, Székely C (2014) Description of raabeia, synactinomyxon and neoactinomyxum developing stages of myxosporeans (Myxozoa) infecting *Isochaetides michaelseni* lastôckin (Tubificidae) in Lake Balaton and Kis-Balaton water reservoir. Hung Syst Parasitol 88:245–259. 10.1007/s11230-014-9496-1 24935127

[CR6] Braun SD (2017) Myxosporean infections of common carp (*Cyprinus carpio*). PhD thesis, University of Veterinary Medicine, Budapest, Hungary

[CR7] Colunga-Ramírez G, Suhaimi NS, Cech G, Molnár K, Székely C, Sellyei B (2024) Morphological and molecular characterisation of two closely related species: *Myxobolus tihanyensis* n. sp. and *Myxobolus sandrae* Reuss, 1906. Int J Parasitol Parasites Wildl 23:100909. 10.1016/j.ijppaw.2024.100909 PMC10873720 38371736

[CR8] Diamant A, Whipps CM, Kent ML (2004) A new species of Sphaeromyxa (Myxosporea: Sphaeromyxina: Sphaeromyxidae) in devil firefish, *Pterois miles* (Scorpaenidae), from the northern Red Sea: Morphology, Ultrastructure, and Phylogeny. J Parasitol 90:1434–1442. 10.1645/GE-336R 15715240

[CR9] Dyková I, Tyml T, Fiala I, Lom J (2007) New data on *Soricimyxum fegati* (Myxozoa) including analysis of its phylogenetic position inferred from the SSU rRNA gene sequence. Folia Parasitol 54(4):272 10.14411/fp.2007.035 18303768

[CR10] El-Mansy A, Székely C, Molnar K (1998a) Studies on the occurrence of actinosporean stages of fish myxosporeans in a fish farm of Hungary, with the description of triactinomyxon, raabeia, aurantiactinomyxon and neoactinomyxon types. Acta Vet Hung 46:259–284 9704528

[CR11] El-Mansy A, Székely C, Molnár K (1998b) Studies on the occurrence of actinosporean stages of myxosporeans in Lake Balaton, Hungary, with the description of triactinomyxon, raabeia and aurantiactinomyxon types. Acta Vet Hung 46:437–450 9713145

[CR12] El–Matbouli M, Hoffmann RW, Mandok C (1995) Light and electron microscopic observations on the route of the triactinomyxon-sporoplasm of *Myxobolus cerebralis* from epidermis into rainbow trout cartilage. J Fish Biol 46:919–935. 10.1111/j.1095-8649.1995.tb01397.x

[CR13] Erséus C, Envall I, De Wit P, Gustavsson LM (2017) Molecular data reveal a tropical freshwater origin of Naidinae (Annelida, Clitellata, Naididae). Mol Phylogenet Evol 115:115–127 10.1016/j.ympev.2017.07.016 28743643

[CR15] Eszterbauer E, Székely C (2004) Molecular phylogeny of the kidney- parasitic *Sphaerospora renicola* from common carp (*Cyprinus carpio*) and *Sphaerospora* sp. from goldfish (*Carassius auratus auratus*). Acta Vet Hung 52:469–478 10.1556/AVet.52.2004.4.9 15595280

[CR14] Eszterbauer E, Marton S, Rácz OZ, Letenyei M, Molnár K (2006) Morphological and genetic differences among actinosporean stages of fish-parasitic myxosporeans (Myxozoa): difficulties of species identification. Syst Parasitol 65:97–114. 10.1007/s11230-006-9041-y 16676228

[CR16] Fiala I (2006) The phylogeny of Myxosporea (Myxozoa) based on small subunit ribosomal RNA gene analysis. Int J Parasitol 36:1521–1534 10.1016/j.ijpara.2006.06.016 16904677

[CR17] Freeman MA, Kristmundsson A (2018) Studies of *Myxidium giardi* Cépède, 1906 infections in Icelandic eels identifies a genetically diverse clade of myxosporeans that represents the *Paramyxidium* n. g. (Myxosporea: Myxidiidae). Parasites Vectors 11:551. 10.1186/s13071-018-3087-y PMC6198514 30348210

[CR18] Gupta A, Chaudhary A, Tyagi A, Sharma B, Singh HS (2021) Molecular tools for identification and classification of Myxozoan parasites (Cnidaria: Myxosporea) in India: current status. J Appl Nat Sci 13(1):51. 10.31018/jans.v13i1.2451

[CR19] Gusakov V, Dien TD, Tran HQ, Thanh NTH, Huan PT, Ha VT, Dinh CN (2023) An annotated checklist of the main representatives of meiobenthos from inland water bodies of central and southern Vietnam—II—annelid worms (Oligochaeta and Aeolosomatidae). Water 15(12):2273. 10.3390/w15122273

[CR20] Hallett SL, Diamant A (2001) Ultrastructure and small subunit ribosomal DNA sequence of *Henneguya lesteri* n. sp. (Myxosporea), a parasite of sand whiting *Sillago analis* (Sillaginidae) from the coast of Queensland, Australia. Dis Aquat Org 46:197–212. 10.3354/dao046197 11710554

[CR21] Hallett SL, Atkinson SD, El-Matbouli M (2002) Molecular characterization of two aurantiactinomyxon (Myxozoa) phenotypes reveal one genotype. J Fish Dis 25(10):627–631

[CR22] Hallett SL, Hartigan A, Atkinson SD (2015) Myxozoans on the move: dispersal modes, exotic species and emerging diseases. In: Okamura B, Gruhl A, Bartholomew JL (eds) Myxozoan evolution, ecology and development. Springer International Publishing, Cham, pp 343

[CR23] Hettige ND, Hashim RB, Ashaari ZH, Kutty AA, Jamil NR (2021) Multivariate statistical approaches to benthic macroinvertebrates and water quality for farming impact assessment in Selangor River, Malaysia

[CR24] Hillis DM, Dixon MT (1991) Ribosomal DNA: molecular evolution and phylogenetic inference. Q Rev Biol 66:411–453. 10.1086/417338 1784710

[CR25] Holzer AS, Sommerville C, Wootten R (2004) Molecular relationships and phylogeny in a community of myxosporeans and actinosporeans based on their 18S rDNA sequences. Int J Parasitol 34:1099–1111. 10.1016/j.ijpara.2004.06.002 15380681

[CR26] Janiszewska J (1957) Actinomyxidia II: new systematics, sexual cycle, description of new genera and species. Zool Pol 8:3–34

[CR27] Källersjö M, von Proschwitz T, Lundberg S, Eldenäs P, Erséus C (2005) Evaluation of ITS rDNA as a complement to mitochondrial gene sequences for phylogenetic studies in freshwater mussels: an example using Unionidae from north-western Europe. Zool Scr 34:415–424. 10.1111/j.1463-6409.2005.00202.x

[CR28] Kearse M, Moir R, Wilson A, Stones-Havas S, Cheung M, Sturrock S, Cheung M, Sturrock S, Buxton S, Cooper A, Markowitz S, Duran C, Thierer T, Ashton B, Meintjes P, Drummond A (2012) Geneious basic: an integrated and extendable desktop software platform for the organization and analysis of sequence data. Bioinformatics 28:1647–1649. 10.1093/bioinformatics/bts199 PMC3371832 22543367

[CR29] Kent ML, Hedrick RP (1986) Development of the PKX myxosporean in rainbow trout *Salmo gairdneri*. Dis Aquat Organ 1:169–182

[CR30] Koel TM, Kerans BL, Barras SC, Hanson KC, Wood JS (2010) Avian piscivores as vectors for *Myxobolus cerebralis* in the greater Yellowstone ecosystem. Am Fish Soc 139:976–988

[CR31] Køie M, Karlsbakk E, Nylund A (2008) The marine herring myxozoan *Ceratomyxa auerbachi* (Myxozoa: Ceratomyxidae) uses *Chone infundibuliformis* (Annelida: Polychaeta: Sabellidae) as invertebrate host. Folia Parasitol 55:100–104 10.14411/fp.2008.013 18666412

[CR32] Kumar S, Stecher G, Li M, Knyaz C, Tamura K (2018) Mega X: molecular evolutionary genetics analysis across computing platforms. Mol Biol Evol 35:1547–1549 10.1093/molbev/msy096 PMC5967553 29722887

[CR33] Liu Y, Whipps CM, Gu ZM, Zeng LB (2010) *Myxobolus turpisrotundus* (Myxosporea: Bivalvulida) spores with caudal appendages: investigating the validity of the genus *Henneguya* with morphological and molecular evidence. Parasitol Res 107(3):699–706. 10.1007/s00436-010-1924-9 20512504

[CR34] Liu XH, Batueva MD, Zhao YL, Zhang JY, Zhang QQ, Li TT, Li AH (2016) Morphological and molecular characterisation of *Myxobolus pronini* n. sp. (Myxozoa: Myxobolidae) from the abdominal cavity and visceral serous membranes of the gibel carp *Carassius auratus gibelio* (Bloch) in Russia and China. Parasit Vectors 9:1–11 10.1186/s13071-016-1836-3 PMC5080772 27782848

[CR35] Lom J, Dyková I (2006) Myxozoan genera: definition and notes on taxonomy, life-cycle terminology and pathogenic species. Folia Parasitol 53:1–36. 10.14411/fp.2006.001 16696428

[CR36] Lom J, McGeorge J, Feist SW, Morris D, Adams A (1997) Guidelines for the uniform characterisation of the actinosporean stages of parasites of the phylum Myxozoa. Dis Aquat Org 30(1):1–9

[CR37] Marcucci C, Caffara M, Goretti E (2009) Occurrence of actinosporean stages (Myxozoa) in the Nera River system (Umbria, central Italy). Parasitol Res 105(6):1517–1530. 10.1007/s00436-009-1586-7 19690888

[CR38] Marques A (1984) Contribution àla connaissance des Actinomyxidies: ultrastructure, cycle biologique, systématique. PhD thesis, Universite´ des Sciences et Techniques de Languedoc, Montepellier, France

[CR39] Martin PJ, Lagnika M, Sonet G, Ibikounle M (2017) DNA barcoding and diversity of groundwater oligochaetes in Benin (West Africa). Genome 60(11):971–972

[CR40] McGeorge J, Sommerville C, Wootten R (1997) Studies of actinosporean myxozoan stages parasitic in oligochaetes from the sediments of a hatchery where Atlantic salmon harbour *Sphaerospora truttae* infection. Dis Aquat Org 30(2):107–119. 10.3354/dao030107

[CR41] Milanin T, Atkinson SD, Silva MR, Alves RG, Maia AA, Adriano EA (2017) Occurrence of two novel actinospore types (Cnidaria: Myxosporea) in Brazilian fish farms, and the creation of a novel actinospore collective group. Seisactinomyxon Acta Parasitol 62(1):121–128 10.1515/ap-2017-0014 28030351

[CR42] Milanin T, Atkinson SD, Silva MRM, Alves RG, Tavares LER, Ribeiro AM, Maia AAM (2018) Occurrence of two novel actinospore types (Cnidaria: Myxozoa) in fish farms in Mato Grosso do Sul state, Brazil. Parasitol Res 117:1757–1764. 10.1007/s00436-018-5856-0 29713902

[CR43] Neetu N, Chauhan DK, Sangeeta Pal SP (2018) Triactinomyxon (actenosporean stage, myxozoa) from fresh waters of Meerut region. J Nat Resour Dev 13:65–68

[CR44] Ohtaka A, Narita T, Kamiya T, Katakura H, Araki Y, Chhay R, Tsukawaki S (2011) Composition of aquatic invertebrates associated with macrophytes in Lake Tonle Sap, Cambodia. Limnology 12:137–144. 10.1007/s10201-010-0330-4

[CR45] Ohtaka A, Sudarso Y, Wulandari L (2015) Records of ten freshwater oligochaete species (Annelida, Clitellata) from Sumatra, Java and Kalimantan, Indonesia. Treubia 34(1):37–57. 10.14203/treubia.v34i0.462

[CR46] Oumouna M, Hallett S, Hoffmann R, El-Matbouli M (2003) Seasonal occurrence of actinosporeans (Myxozoa) and oligochaetes (Annelida) at a trout hatchery in Bavaria. Ger Parasitol Res 89(3):170–184. 10.1007/s00436-002-0683-7 12541058

[CR47] Özer A, Wootten R (2000) The life cycle of *Sphaerospora truttae* (Myxozoa: Myxosporea) and some features of the biology of both the actinosporean and myxosporean stages. Dis Aquat Organ 40:33–39. 10.3354/dao 10785861

[CR48] Özer A, Wootten R (2001) Release of actinosporean and myxosporean spores from their hosts, with special reference to both stages of *Sphaerospora truttae* (Myxozoa, Myxosporea). Acta Parasitol 46(2):103–112

[CR49] Özer A, Wootten R, Shinn AP (2002) Survey of actinosporean types (Myxozoa) belonging to seven collective groups found in a freshwater salmon farm in Northern Scotland. Folia Parasitol 49:189–210 10.14411/fp.2002.036 12418811

[CR50] Rambaut A (2018) FigTree V. 1.4.4: Tree Figure Drawing Tool. http://tree.bio.ed.ac.uk/software/figtree. (Accessed 10 October 2025)

[CR51] Rangel LF, Rocha S, Castro R, Severino R, Casal G, Azevedo C, Cavaleiro F, Santos MJ (2015) The life cycle of *Ortholinea auratae* (Myxozoa: ortholineidae) involves an actinospore of the triactinomyxon morphotype infecting a marine oligochaete. Parasitol Res 114:2671–2678. 10.1007/s00436-015-4472-5 25899324

[CR52] Rangel LF, Castro R, Rocha S, Cech G, Casal G, Azevedo C, Szkéley C, Cavaleiro F, Santos MJ (2016) Description of new types of sphaeractinomyxon actinospores (Myxozoa: myxosporea) from marine tubificid oligochaetes, with a discussion on the validity of the tetraspora and the endocapsa as actinospore collective group names. Parasitol Res 115:2341–2351. 10.1007/s00436-016-4983-8 26969178

[CR53] Rocha S (2023) Synopsis of the aurantiactinomyxon collective group (Cnidaria, Myxozoa), with a discussion on the validity of morphotype definition and demise of guyenotia. Syst Parasitol 100:307–323. 10.1007/s11230-023-10089-1 PMC10129985 37060426

[CR54] Rocha S, Alves Ǎ, Fernandes P, Antunes C, Azevedo C, Casal G (2019a) New actinosporean description prompts union of the raabeia and echinactinomyxon collective groups (Cnidaria, Myxozoa). Dis Aquat Org 135:175–191. 10.3354/dao03389 31486410

[CR55] Rocha S, Alves Â, Antunes C, Azevedo C, Casal G (2019b) Molecular data infers the involvement of a marine aurantiactinomyxon in the life cycle of the myxosporean parasite *Paramyxidium giardi* (Cnidaria, Myxozoa). Parasitology 146(12):1555–1563 10.1017/S0031182019000866 31232243

[CR56] Rocha S, Alves Â, Antunes C, Rodrigues P, Casal G (2024) Characterization of novel aurantiactinomyxon types (Cnidaria, Myxosporea) from the oligochaete *Ilyodrilus templetoni* (Southern, 1909), with a comprehensive phylogeny of the collective group. J Invertebr Pathol 203:108043. 10.1016/j.jip.2023.108043 38104963

[CR57] Rodrigues LFT, Rosa BFJV, Lobo H, Divino AC, Da Gama Alves R (2015) Diversity and distribution of oligochaetes in tropical forested streams, Southeastern Brazil. J Limnol 74(3):433–443

[CR58] Rosser TG, Griffin MJ, Quiniou SM, Greenway TE, Khoo LH, Wise DJ, Pote LM (2014) Molecular and morphological characterization of myxozoan actinospore types from a commercial catfish pond in the Mississippi Delta. J Parasitol 100:828–839. 10.1645/13-446.1 25003942

[CR59] Suhaimi NS, Sellyei B, Cech G, Székely C, Borkhanuddin MH (2024a) First record and description of actinospore stages (raabeia, triactinomyxon, and aurantiactinomyxon types) of fish parasitic myxozoans from Malaysia. Int J Parasitol Parasites Wildl 24:100964. 10.1016/j.ijppaw.2024.100964 PMC11298608 39105042

[CR60] Suhaimi NS, Sellyei B, Udvari Z, Székely C, Cech G (2024b) Characterization of four novel actinospore types of fish parasitic myxozoans and the occurrence of *Branchiodrilus hortensis* and *Ophidonais serpentina* from fish farms of Hungary. Int J Parasitol Parasites Wildl 25:100994 10.1016/j.ijppaw.2024.100994 PMC11497487 39445088

[CR61] Suhaimi NS, Székely C, Sellyei B, Cech G (2025) Actinosporean diversity in a Hungarian fish farm and description of the life cycle of *Zschokkella chezhachei*. Int J Parasitol Parasites Wildl 101124. 10.1016/j.ijppaw.2025.101124 PMC12355032 40822575

[CR62] Suhaimi NS, Sellyei B, Mosonyi-Borzák R, Cech G, Molnár K, Székely C (2026) *Thelohanellus* species in common carp (*Cyprinus carpio*) and their life cycles from fish farms and Lake Balaton in Hungary. Aquat Rep 46:103380. 10.1016/j.aqrep.2026.103380

[CR63] Székely C, Sitja-Bobadilla A, Alvarez-Pellitero P (2000) First report on the occurrence of an actinosporean stage (Myxozoa) in oligochaetes from Spanish freshwaters. Acta Vet Hung 48(4):433–441. 10.1556/004.48.2000.4.6 11402660

[CR64] Székely C, Yokoyama H, Urawa S, Timm T, Ogawa K (2003) Description of two new actinosporean types from a brook of Fuji Mountain, Honshu, and from Chitose river, Hokkaido, Japan. Dis Aquat Org 53:127–132. 10.3354/dao053127 12650245

[CR65] Székely C, Avenant-Oldewage A, Molnar K (2004) Description of a new actinosporean type from South African freshwaters. Dis Aquat Organ 61(1–2):95–102. 10.3354/dao061095 15584416

[CR66] Székely C, Borkhanuddin MH, Cech G, Kelemen O, Molnár K (2014) Life cycles of three *Myxobolus* spp. from cyprinid fishes of Lake Balaton, Hungary involve triactinomyxon-type actinospores. Parasitol Res 113:2817–2825. 10.1007/s00436-014-3942-5 24828348

[CR67] Thompson JD, Higgins DG, Gibson TJ (1994) Clustal W: improving the sensitivity of progressive multiple sequence alignment through sequence weighting, position-specific gap penalties and weight matrix choice. Nucleic Acids Res 22:4673–4680. 10.1093/nar/22.22.4673 PMC308517 7984417

[CR68] Timm T (1999) Naturalist Handbooks I: A Guide to the Estonian Annelida. Estonian Academic

[CR69] Vineetha S, Bijoy Nandan S, Rakhi Gopalan KP (2015) Comparative study on macrobenthic community structure with special reference to oligochaetes during drought and flooded phases in a Tropical Kole Wetland, India. Int J Mar Sci 5(41):1–10. 10.5376/ijms/2015.05.0041

[CR70] Whipps CM, Adlard RD, Bryant MS, Kent ML (2003) Two unusual myxozoans, *Kudoa quadricornis* n. sp. (Multivalvulida) from the muscle of godspotted trevally (*Carangoides fulvoguttatus*) and *Kudoa permulticapsula* n. sp. (Multivalvulida) from the muscle of Spanish mackerel (*Scomberomorus commersoni*) from the Great Barrier Reef, Australia. J Parasitol 89:168–173 10.1645/0022-3395(2003)089[0168:TUMKQN]2.0.CO;2 12659322

[CR71] Whipps CW, Atkinson SD, Hoeksema BW (2025) World List of Myxozoa. Accessed at https://www.marinespecies.org/myxozoa on 2025-10-02

[CR72] White TJ, Bruns TD, Lee S, Taylor JW (1990) Amplification and direct sequencing of fungal ribosomal RNA genes for phylogenetics. In: Innis MA, Gelfand DH, Sninsky JJ, White TJ (eds) PCR Protocols: A Guide to Methods and Applications. Academic, London, pp 315–322

[CR73] Xi BW, Zhang JY, Xie J, Pan LK, Xu P, Ge XP (2013) Three actinosporean types (Myxozoa) from the oligochaete *Branchiura sowerbyi* in China. Parasitol Res 112:1575–1582 10.1007/s00436-013-3306-6 23377120

[CR74] Xi BW, Zhou ZG, Xie J, Pan LK, Yang YL, Ge XP (2015) Morphological and molecular characterization of actinosporeans infecting oligochaete *Branchiura sowerbyi* from Chinese carp ponds. Dis Aquat Org 114:217–228. 10.3354/dao02859 26036829

[CR75] Xiao CX, Desser SS (1998) Actinosporean stages of myxozoan parasites of oligochaetes from Lake Sasajewun, Algonquin Park, Ontario: New forms of echinactinomyxon, neoactinomyxum, aurantiactinomyxon, guyenotia, synactinomyxon and antonactinomyxon. J Parasitol 84(5):1010–1019. 10.2307/3284635 9794645

[CR76] Zhao DD, Borkhanuddin MH, Wang W, Liu Y, Cech G, Zhai Y, Székely C (2016) The life cycle of *Thelohanellus kitauei* (Myxozoa: Myxosporea) infecting common carp (*Cyprinus carpio*) involves aurantiactinomyxon in *Branchiura sowerbyi*. Parasitol Res 115:4317–4325. 10.1007/s00436-016-5215-y 27492197

