# Supplementary material for: Description of three new types of aurantiactinomyxon actinospores (Myxozoa: Myxosporea) from the oligochaete Aulophorus sp. in Tasik Telabak, Malaysia
Source: Parasitol Res. 2026 Jul 29;125(1):134. doi: 10.1007/s00436-026-08739-6 (PMC13710626; doi:10.1007/s00436-026-08739-6)
Supplement: Supplementary file 1 [file 436_2026_8739_MOESM2_ESM.docx]

**Supplementary Table 1.** Morphometric data from additional infections of Aurantiactinomyxon type 3 (two actinospores measured per host), including spore body, caudal processes, and polar capsules. All measurements are in μm and ’–’ indicates no data. SBD: actinospore body diameter, CPL: caudal processes length, CPW: caudal processes width, PCL: polar capsule length, PCW: polar capsule width.

| **Individual** | **SBD** | **CPL** | **CPW** | **PCL** | **PCW** |
| --- | --- | --- | --- | --- | --- |
| 1 | 11.5 ± 0.3 | 47.7 ± 0.6 | 6.3 ± 0.2 | 3.1 ± 0.2 | 2.0 ± 0.1 |
| 2 | 11.2 ± 0.5 | 43.9 ± 4.0 | 6.3 ± 0.4 | 3.3 ± 0.0 | 2.0 ± 0.1 |
| 3 | 12.1 ± 1.7 | 44.7 ± 2.8 | 6.3 ± 0.3 | 3.4 ± 0.1 | 1.9 ± 0.2 |
| 4 | 12.0 ± 1.6 | 46.8 ± 0.0 | 6.3 ± 0.0 | 3.3 ± 0.2 | 2.1 ± 0.1 |
| 5 | 11.4 ± 1.1 | 40.7 ± 0.3 | 6.7 ± 0.1 | 3.5 ± 0.1 | 2.2 ± 0.2 |
| 6 | 12.1 ± 0.5 | 42.8 ± 3.3 | 6.4 ± 0.5 | 3.5 ± 0.0 | 2.2 ± 0.1 |
| 7 | 11.9 ± 1.9 | 41.8 ± 1.6 | 6.4 ± 0.5 | 3.5 ± 0.0 | 2.1 ± 0.4 |
| 8 | 11.9 ± 0.2 | 48.0 ± 1.8 | 5.9 ± 0.1 | 3.3 ± 0.2 | 1.9 ± 0.1 |
| 9 | 12.1 ± 0.1 | 44.8 ± 0.1 | 6.4 ± 0.5 | 3.1 ± 0.0 | 1.8 ± 0.2 |
| 10 | 12.1 ± 0.3 | 45.7 ± 0.1 | 6.0 ± 0.7 | 3.0 ± 0.1 | 1.8 ± 0.1 |
| 11 | 11.7 ± 0.0 | 43.0 ± 1.8 | 6.1 ± 0.6 | 3.2 ± 0.1 | 2.1 ± 0.1 |
